# Plasma levels of interlukin-4 and Interferon-γ in patients with chronic or healed cutaneous leishmaniasis

**Published:** 2014-03

**Authors:** Ahmad Reza Taheri, Vahid Mashayekhi Goyonlo, Yalda Nahidi, Nasrin Moheghi, Jalil Tavakkol Afshari

**Affiliations:** 1 Cutaneous Leishmaniasis Research Center, Emam Reza Hospital, School of Medicine, Mashhad University of Medical Sciences, Mashhad, Iran; 2 Immunology Research Center, Mashhad University of Medical Sciences, Mashhad, Iran

**Keywords:** Cutaneous leishmaniasis, Interferon-γ, Interlukin-4, T helper cell

## Abstract

***Objective(s):*** In this study, the serum level of interferon-γ (IFN- γ) and interlukin-4 (IL-4) was evaluated as a marker of Th1 and Th2 immune response that influence the clinical course of cutaneous leishmaniasis .

***Materials and Methods:*** This cross-sectional study was conducted on 44 cases of cutaneous leishmaniasis (21 cases with healed lesions and 23 cases with chronic non-healing lesions. Thirty-two non-infected persons living in the area were considered as controls. Serum levels of IFN- γ and IL-4 were determined using ELISA, and the results along with clinical data were analyzed using SPSS 11.5.

***Results:*** Serum IFN-γ level was not significantly different between various patient groups and control (*P*=0.27), but the serum level of IL-4 in patient groups was higher than in healthy subjects, and it was higher in patients with non-healed chronic cutaneous leishmaniasis than those with healed lesions (*P*<0.01).

***Conclusion:*** Serum IL-4 level is a good marker for evaluation of the clinical course of cutaneous leishmaniasis.

## Introduction

*Leishmania*, an intracellular parasite, causes a wide range of cutaneous lesions from a single ulcer with spontaneous healing tendency to chronic, slow- or non-healing skin lesions (1). Cutaneous leishmaniasis (CL) is the most important parasitic disease in Iran. Mashhad a major city in the North-East region of Iran is a well-known zone of this infection (2). Despite treatment with the standard drug, meglumine antimoniate, some cases do not recover completely and become chronic.

It seems that various clinical features and course of this disease are related to the host immune system. Immune response against intracellular parasites is dependent on the balance between Th1 and Th2 cells (3). IFN-γ is a Th1 cytokine that plays a role in controlling the infection and killing the parasites by Enhancing macrophages activity (4-7). IL-4 derived from Th2 inhibits the proliferation of Th1 and IFN-γ production and consequently the parasites escape from host immune system and ultimately the disease becomes chronic (4, 6).

Most studies on the pathogenesis of CL have been conducted on L. major induced leishmaniasis in mouse models. The cytokines have been measured after experimental stimulation of lymphoid cells by the parasite, which do not necessarily reflect the in vivo immune response..  As well, studies on the human and *in vivo* expriments are often conducted by isolating the peripheral blood mononuclear cells and measuring the cytokines after the exposure of these cells to *Leishmania* antigens in culture that are costly and time consuming. On the other hand, measurement of these cytokines by easier methods could be useful in prediction of prognosis and choosing the appropriate treatment at early stages of the disease considering the cell profile and cytokines.

We investigated the serum levels of IFN-γ and IL-4 in patients with healed and chronic lesions of CL using enzyme-linked immunosorbent assay (ELISA) and compared the results with other studies.

## Materials and Methods

This cross-sectional study was conducted in patients with CL referred to Emam Reza Hospital in Mashhad, Iran during the year 2006. The patients included 21 individuals (44.7%) with healed scar of previous CL, 23 individuals (48.9%) with chronic lesions of CL (no improvement within 2 years from the onset) and 3 individuals (6.4%) with both healed and chronic lesions. Since the number of cases with simultaneous scar and lesion was too small (only 3 cases) to be statistically analyzed and utilized, it was removed from this study. Thirty-two healthy cases without previous history of CL were enrolled as controls.

**Table 1:** Epidemiologic data of patients with cutaneous leishmaniasis referred to Leishmaniasis clinic of Imam Reza Hospital, Mashhad, Iran

Leishmaniasis type		Healed lesions with scar Patients (%)	Chronic lesion persisting more than two years Patients (%)	Total
Frequency		21(44.7)	23(48.9)	44(100%)
Gender	Male	10(47.6)	9(39.1)	194(2.6%)
	Female	11(52.4)	14(60.9)	25(57.4%)
Age average		30.65 ± 18.60	27.13 ± 19.76	29.73 ± 19.05

The written consent was obtained from all patients before entering the study. Five ml of peripheral blood from patients and healthy (or control) subjects were obtained and the serum was separated immediately after clot formation. The serum was transferred into separate tube and kept at -20° C in a freezer at - 20°C and then at - 70°C. ELISA was performed using the kits of Bender Medsystems Inc. (Vienna, Austria) in accordance with company guidelines. Kits of human IFN-γ (BMS228) and human IL-4 (BMS225 / 2) were used for assessment of IFN-γ and IL-4, respectively. Optical absorption at the corresponding wavelength was read by ELISA reader (Awareness Technology Inc, USA). The concentration was calculated using a standard curve and blank well. These results along with demographic and clinical information were analyzed using SPSS 11.5 software and tests of T-test, Mann-Whitney and Kruskal-Wallis. The difference in mean values was considered significant when the *P-*value was <0.05.

## Results

Epidemiologic data of the patients is summarized in Table 1. Different groups of patients had no significant difference in age (*P*=0.132), gender (*P*=0.80) and number of lesions (*P*=0.19). 

According to the non-normal distribution of IFN-γ and IL-4 values, the nonparametric Kruskal-Wallis test was used to compare values between different groups.

 In spite of obviously different levels of IFN-γ in studied cases, the serum IFN–γ value was not significantly different between groups (*P*=0.27). The serum value of IL-4 showed significant differences between various groups (*P*<0.01), so that the serum level of IL-4 was higher in patients than control, and those with chronic CL lesions had the highest level of IL-4 among patients.

In order to compare IFN-γ and IL-4 values within the groups, nonparametric Mann-Whitney test was used, with *P*-values of 0.84 and 0.005 for IFN-γ and IL-4 in patients with healed lesions with scarring and those with chronic lesions, respectively. 

Comparison of IFN-γ and IL-4 values between patients with healed lesions with scarring and control group showed the *P*-values of 0.80 and <0.01, respectively, so, IL-4 value was significantly lower in cured patients than healthy subjects. 

*P*-values for the differences of IFN-γ and IL-4 levels between patients with chronic lesions and control group, were 0.30 and <0.01, respectively, so IL-4 was significantly higher in patients with chronic lesions than healthy individuals. 

## Discussion

Clinical features of leishmaniasis depend on host immunity and genetic, parasite species, type of vector and environmental factors (3, 7). Generally, skin lesions of Old World CL are self limited, but chronic and non-healing forms have been described. Non-healing lesions of *L. major* and *L. tropica*   induced lesions are considered as chronic non-healing CL when there is no improvement after 1 and 2 years, respectively (12, 13). 

According to experimental studies in mice models, it is known that susceptibility of BALB/c and resistance of C57BL/6 mice to *L. major* infection are related to predominant Th2 ( with production of IL-4 and IL-10) or Th1 ( early and continued production of IFN-γ) immune response to *Leishmania* antigen, respectively (3, 7, 8, 9, 10, 11). IFN- γ is a major Th1 derived cytokine that plays essential role in controlling Leishmania infection (7). 

**Table 2:** Serum levels of IFN-γ and IL-4 in different groups of patients and controls

Serum evaluation result group		IFN-γ		IL-4	
		Sample number	Value (ng/ml)	Sample number	Value (ng/ml)
Cutaneous leishmaniasis patients	Healed scar	18	5.88 ± 7.08	17	2.65 ± 4.75
	Chronic lesions	19	50.078±130.53	19	54.41±187.39
	Simultaneous presence of scar and lesion	3	1.86 ± 1.61	3	1.40 ± 2.17
Control		32	3.74 ± 0.99	31	54.75 ± 14.1
Results (PV)		0.27		<0.01	

Th2 cells that produce IL-13, IL-10, IL-5 and IL-4, are often engaged with B cells differentiation and maturation with consequence of antibodies production (14). IL-4 inhibits Th1 cells proliferation and IFN- γ production, so parasite could escape from host immunity (7). However, early production of IL-4 does not necessarily correlate with susceptibility to *L. major* as resistant C57BL/6 mice also produce this cytokine initially until infection resolves with the development of Th1 response (6).

In a study by Castellano *et al*, production of cytokines in culture supernatants of blood T cells and specific antibody isotypes plasma levels were analyzed in 20 patients with active CL, 30 individuals with cured CL, and 34 individuals without lesions living in the same endemic area as control group. They found significantly higher levels of IFN-γ in patients groups (either with active or cured lesions) in comparison to control group and also IL-4 levels were higher in patients with active lesions than in cases with healed lesions or control group (7). Ajdary *et al* showed that IFN-γ was not significantly different between early and persisted CL patients but it was higher in comparison to control group. IL-5 was significantly higher in cases of non-healing CL than both early CL and control group. The chronic CL cases demonstrated also higher levels of IL-13. They concluded that chronic non-healing CL shows a mix pattern of Th1 and Th2 responses whereas early CL presents a Th1 predominant response (12). Also in our study, the higher levels of Th2 related cytokine (IL-4) were detected in chronic non-healing cases but contrasting to Ajdary *et al* data, we did not find significant difference between serum IFN- γ level between *Leishmania* patients and control group. 

In a study by Meimandi *et al*, 6 cases of untreated dry type CL with less than 2 years duration (defined as acute) and 3 cases of CL persisted for more than 2 years (defined as chronic non lupoid) and 3 cases of CL presented with cherry red papules around previous CL scar (lupoid or recidivans type of CL) were biopsied and specimens were studied by immunohistochemical staining for Th1 and Th2 markers. They found that, IL-4 (Th2 marker) in chronic non lupoid and IFN-γ (Th1 marker) in acute and lupoid lesions are significantly pronounced (14). In another study published in the year 2000 by Ajdary *et al*, peripheral blood mononuclear cells of CL cases were incubated with *L. major* antigens. Twenty two cases of active wet form, 17 cases of non-healing chronic lesions and 17 cases with healed scaring CL were included in their study. They found that active and healed scaring CL had higher amounts of IFN- γ in comparison to cases with non-healing chronic lesions. In the latter group, production of IFN- γ by stimulated peripheral blood mononuclear cells was very low or undetectable but their mononuclear cells could produce higher amounts of IL-4 (15). 

Most studies on immunopathogenesis of leishmaniasis have been conducted in experimental models and they have evaluated cytokine production of lymphocytes *in vitro* using *L. major* as stimulating antigen for lymphoid cells. These studies could not necessarily reflect *in vivo* immune response of infected host (3). Cytokines are key elements of immune responses and represent immunity orientation and competency *in vivo* or *in vitro*. So, we tried to use ELISA to evaluate IL-4 and IFN-γ levels in peripheral blood of our cases and control group. IFN-γ did not show statistically significant difference between case and control group. Serum levels of IL-4 in comparison to control group, it was significantly higher in cases with chronic lesion, but in cured patients, IL-4 showed lower levels in peripheral blood, statistically different from control. 

Our findings might be explained as follows: 

1) Th2 activity in chronic leishmaniasis is associated with higher levels of IL-4 whereas in healed lesions and control group, there is no reason for Th2 activity. On the other hand, IFN-γ secretion would not be induced in chronic leishmaniasis because of the Th2 predominant response so that it is expected not to be statistically different from cases with healed lesions - IFN-γ has no more stimulatory signals- or from control group.

2) Probably serum levels of IFN-γ do not reflect its participation in localized inflammatory conditions like CL. 

## Conclusion

Regarding previous studies, our results of IL-4 levels in different groups could be expected but we did not found significant difference in the levels of IFN-γ between two groups of CL cases and also in comparison to control group. 
